# Ultra-Widefield Fluorescein Angiography Image Brightness Compensation Based on Geometrical Features

**DOI:** 10.3390/s22010012

**Published:** 2021-12-21

**Authors:** Wojciech Więcławek, Marta Danch-Wierzchowska, Marcin Rudzki, Bogumiła Sędziak-Marcinek, Slawomir Jan Teper

**Affiliations:** 1 Faculty of Biomedical Engineering, Silesian University of Technology, Roosevelta St. 40, 41-800 Zabrze, Poland; marta.danch-wierzchowska@polsl.pl (M.D.-W.); marcin.rudzki@polsl.pl (M.R.); 2 Clinical Department of Ophthalmology, Faculty of Medical Sciences in Zabrze, Medical University of Silesia, Panewnicka St. 65, 40-760 Katowice, Poland; bogsedziak@gmail.com (B.S.-M.); slawomir.teper@sum.edu.pl (S.J.T.)

**Keywords:** UWFA, FA, image enhancement, gamma correction, curve length, solid angle, ophthalmology, image processing

## Abstract

Ultra-widefield fluorescein angiography (UWFA) is an emerging imaging modality used to characterise pathologies in the retinal vasculature, such as microaneurysms (MAs) and vascular leakages. Despite its potential value for diagnosis and disease screening, objective quantitative assessment of retinal pathologies by UWFA is currently limited because laborious manual processing is required. In this report, we describe a geometrical method for uneven brightness compensation inherent to UWFA imaging technique. The correction function is based on the geometrical eyeball shape, therefore it is fully automated and depends only on pixel distance from the center of the imaged retina. The method’s performance was assessed on a database containing 256 UWFA images with the use of several image quality measures that show the correction method improves image quality. The method is also compared to the commonly used CLAHE approach and was also employed in a pilot study for vascular segmentation, giving a noticeable improvement in segmentation results. Therefore, the method can be used as an image preprocessing step in retinal UWFA image analysis.

## 1. Introduction

Fluorescein angiography was introduced as a diagnostic tool over 50 years ago. Because of its constant development, it still remains a reliable diagnostic tool for many retinal diseases, i.e., diabetic retinopathy, retinal vein occlusion, age-related macular degeneration retinopathy, uveitis, or retinal/choroidal dystrophy [1,2]. The literature says that first pathological changes occur in the peripheral area of the retina and, as the disease progresses, cover an area increasingly larger towards the center. Retinal imaging methods used so far have included about 30–50${}^{\circ }$ of the retina. It turns out that the depicted area is too narrow to recognize the early symptoms of retinal degeneration [3]. In recent years three approaches have been introduced to expand the visible retina area: (1) assembly of traditional angiograms, i.e., several images and at least two acquisition protocols; (2) adding an additional lens to the optical path of the classic camera; (3) introduction of new devices enabling observation of a wider area of the retina [3,4]. Ultra-wide field fluorescein angiography (UWFA) is an extension of the third group of methods and enables imaging of a significantly larger retinal area (with angle over 200${}^{\circ }$) [1]. Comparing the UWFA with a classic fundus camera, the obtained $200^{\circ }$ from the center of the eye corresponds to a conventional angle measure of approximately $125^{\circ }$ [2].

The optical system, as well as the software processing UWFA images after the acquisition, are still sources of some issues that need to be improved. The associated deformations of the peripheral area may disrupt the effects of the quantitative analysis of those areas by artificially increasing the obtained values. Mapping and optimization of the three-dimensional spherical surface onto a flat image are one of the key transformations performed during the acquisition [5,6,7]. Another issue is the wavelength of excitation light and the barrier filter used, specific for each device. The images obtained by different devices may contain inherent differences, especially when evaluating the fovea. However, previous studies indicate that these differences have a minor impact when estimating the peripheral area of the eye [2].

The vital issue, which needs to be addressed during UWFA images processing, is the uneven brightness of the image. The further away from the central area, the darker the image and the lower the contrast are. This resembles the problem of bias correction known in other imaging modalities [8]. Uneven brightness compensation during image preprocessing seems crucial in retinal qualitative and quantitative image analysis. In the case of retinal image analysis, one of the latest tasks is retinal vessel segmentation. Image brightness correction sometimes is omitted in processing methods that utilize deep learning, for example, convolutional neural networks [9,10,11,12]. There are also reports where this step is skipped even in the “traditional” image processing algorithms [13,14]. However, only one of the aforementioned works is based on UWFA images, while others are based on standard retina images. Therefore abandoning the preprocessing may not always be possible for UWFA images.

The inhomogeneous brightness of retinal angiographic images stems from the geometry of the imaged structure, therefore the transformations most often applied at the preprocessing stage should be local in nature, depending on regional image properties. One such transformation, very often applied to retinal angiographic images, is the technique of Contrast Limited Adaptive Histogram Equalization (CLAHE) [15], which differs from ordinary histogram equalization by local parameter selection. Sopharak et al. [16] use this method as a preprocessing preceding microaneurysms detection. The CLAHE algorithm plays a similar role in [17]. A more comprehensive approach is introduced in [18], where the CLAHE algorithm is also used because the authors claim that CLAHE provides the utmost improvement in the prediction accuracy. CLAHE algorithm is also used during quantification of optical coherence tomography angiography images [19], and also as one of the processing steps in de-noising and contrast enhancement of retinal fundus images [20,21]. Several other attempts have also been made in brightness compensation methods to improve the segmentation accuracy in retinal imaging, e.g., homomorphic filtering function with Gaussian high-pass filter [22] or N4-bias field correction [23]. Those works, however, focus on narrow-field retinal angiograms, not UWFA, where the effect of low brightness in the periphery is not that significant.

The main goal of this work is to develop a brightness correction method, especially for UWFA images, that enhances the peripheral retinal areas without causing disruptions in the central regions. To achieve this goal, we propose an automated gamma correction procedure. The gamma value for this procedure is computed locally based on the geometrical conditions resulting from the image acquisition technique. Our method is validated on 256 images, taking into consideration the image acquisition phases (early, mid, late) and exhibiting physiological and pathologic features (leakages, ischaemia, microaneurysms) in patients diagnosed with diabetic retinopathy. Several image quality measures are calculated for the central and peripheral image regions, showing that the method introduces negligible image distortion in central areas while enhancing the peripheral image regions. The presented procedure may provide new opportunities for detecting, analyzing, and evaluating underlying image features that have important clinical and research applications.

## 2. Materials and Methods

Ultra-widefield fluorescein angiography images require brightness compensation, especially in the peripheral areas. It is inducted by the spherical shape of the retina and properties of the image acquisition technique. We propose a method inspired by the retina shape to overcome these inconveniences. Our proposition is presented in two following steps. The first one is a simplified 2D case, which results in a 1D correction function, and the second one is generalized to the spatial 3D case, which results in a 2D correction function. Both are based on the assumption that the eyeball can be approximated as a sphere, which is often encountered in the retinal image analysis [1,2,5,6,24].

### 2.1. 2D Case Projected onto 1D Space

**Figure 1:**
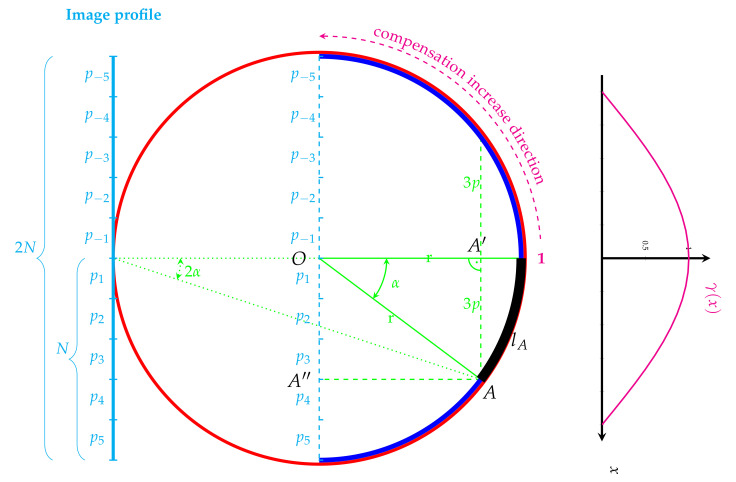
The principle for luminance weights determination in 2D space projected onto 1D.

Consider a central cross-section of an eyeball presented in Figure 1. We assume (similarly to other authors [2,5,7]) that an eyeball can be represented as a sphere. A red circle illustrates the great circle (equator), and a blue semi-circle located on the backside of the eyeball represents the retina. We assume that it spans exactly a half-circle (it covers a straight angle, π or $180^{\circ }$). Outside the eyeball, opposite to the retina, there is an acquisition device, which reconstructs the image. This is indicated by a single image profile (cyan line). It is composed of 2N (2N is a horizontal resolution of an image) pixels $p_{x}$ having spatial size *p* (length in 1D, square side in 2D). This image profile, to simplify further mathematical considerations, is duplicated and is placed inside the eyeball (dotted cyan line in Figure 1).

Based on the observation that in UWFA images, the brightness and contrast fade with the distance from the center, the centrally located pixel does not require brightness correction. Thus, it is processed without changes (i.e., the correction coefficient is 1). Other pixels that express retina points situated farther away from the center require revision that depends on pixel location (represented by the distance from the center). The farther away the pixel from the center is, the more substantial pixel value correction is required. A direction of increasing demand for correction is presented in Figure 1 using a magenta curved arrow.

The proposed principle of correcting factor determination indirectly stems from the geometric distance from the center, measured by the curve length:

(1) $$l_{A}=r\cdot \alpha ,$$

where *r* denotes the radius of the eyeball and α a central angle in radians on which the arc $l_{A}$ is spanned. This angle can be determined based on geometrical relations, in particular it can be determined from a right triangle OAA’:

(2) $$\sin\alpha =\frac{x\cdot p}{r},$$

where *x* is an integer number of pixels between $A^{\prime \prime }$, which is a projection of the currently analysed point *A* onto image profile, and the central axis (green horizontal line) of the eyeball or point *O*. This value is within the range 1…N. The approach is symmetrical for the mentioned central axis. In Figure 1, an exemplary right triangle is shown, with two sides having lengths *r* and 3p (i.e., x=3), respectively.

Moreover, the retina radius *r* can be expressed using the number of pixels as r=N·p, hence:

(3) $$\sin\alpha =\frac{x}{N}.$$

Finally, using the inverse trigonometric function arcsin(•), the curve length can be defined as:

(4) $$l_{A}=r\cdot \arcsin\frac{x}{N}.$$

This distance function is proportional to the angle $\arcsin\frac{x}{N}$ expressed by an integer number of pixels $p_{x}$ measured relative to the central axis. For small alpha α≈0, that is pixels located centrally (x≈0), it gives a value of 0, and for the farthest retina pixels ($\alpha \approx \frac{\pi }{2}$ or $\alpha \approx 90^{\circ }$ that is pixels located peripherally x≈N) it gives a value close to $r\cdot \frac{\pi }{2}$. Moreover, the radius of the eyeball *r* represents a constant scaling factor in this equation. Because we focus solely on the proportions within the eyeball, we assume the eyeball to be a unit sphere, therefore r=1.

In order to adjust these mathematical conditions to the properties of the retinal acquisition technique, we propose the correction function for the 2D case projected onto 1D space to be defined as

(5) $$\gamma \left(x\right)=\cos\left(\arcsin\frac{x}{N}\right).$$

This γ(x) function, as is shown in Figure 1, is decreasing. Its values (in the range 0…1) for a particular *x* are then used as an argument for the gamma image correction stage [25] according to:

(6) $$J\left(N\left(p_{x}\right)\right)=I{\left(N\left(p_{x}\right)\right)}^{\gamma (x)}.$$

that is performed locally for a small neighbourhood (N(•)) of a pixel *p* located at *x*. For γ<1 the resulting intensity rescaling is non-linear and results in contrast enhancement of hypointensive image regions. For γ=1, the rescaling function is linear, thus the image intensities remain unchanged.

### 2.2. 3D Case Projected onto 2D Space

**Figure 2:**
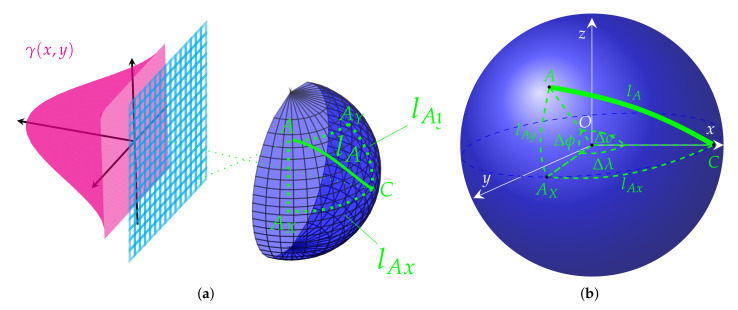
The principle for contrast compensation determination in 3D space: (**a**) spherical mapping of the retina to 2D image domain with correction function γ(x,y), (**b**) great-circle distance.

The method presented above needs a generalization for spatial conditions projected onto the 2D case, as shown in Figure 2. Similarly, the basis for defining the correction function γ(•) is the distance between the central retina point *C* and any other retina element e.g., point *A*. This distance is an arc $l_{A}$ spanned on a sphere.

This curve part is the shortest distance between two points denoted as *C* and *A* on the surface of a sphere, measured along the surface of the sphere. To compute the distance in spaces with curvature, straight lines are replaced with geodesics. Geodesics on the sphere are circles on the sphere whose centers coincide with the center of the sphere and are called great circles or orthodromes. The determination of the great circle distance (or orthodromic distance) is part of a more general problem of great circle navigation, which also computes the azimuths at the end-points and intermediate way-points.

Through any two points on a sphere (*C* and *A* in Figure 2b) that are not directly opposite to each other (i.e., not on a diameter), a unique great circle can be determined. The two points divide the great circle into two arcs. The length of the shorter arc ($l_{A}$ in Figure 2b) is then the great circle distance between these points.

Consider two points *C* and *A* with their geographical longitude $\lambda_{C}$, $\lambda_{A}$ and latitude $\phi_{C}$, $\phi_{A}$ in radians, respectively. Their absolute differences are denoted as Δλ and Δϕ. Then the central angle between them Δσ is given by the spherical law of cosines:

(7) $$\Delta \sigma =\arccos\left(\sin\phi_{A}\sin\phi_{C}+\cos\phi_{A}\cos\phi_{C}\cos\Delta \lambda \right)$$

if one of the poles is used as an auxiliary third point on the sphere ([26], pp. 323–326). Given this angle in radians, the actual arc length $l_{A}$ on a sphere of radius *r* can be trivially computed as:

(8) $$l_{A}=r\;\Delta \sigma .$$

In the case illustrated in Figure 2a the Equation (7) can be simplified, because point *C* is a reference point located centrally with $\lambda_{C}=0$ and $\phi_{C}=0$. Hence, $\sin\phi_{C}=0$ and $\cos\phi_{C}=1$. Moreover, since $\cos\Delta \lambda =\cos\lambda_{A}$, then the arc length in 3D space is given by:

(9) $$l_{A}=r\;\arccos\left(\cos\phi_{A}\cos\lambda_{A}\right).$$

Both angles $\phi_{A}$ and $\lambda_{A}$ can be computed by analogy to the 2D case presented in the previous Section 2.1. Thus, if the image resolution is 2N×2M (where 2N is the width and 2M is the height of the image), then:

(10) $$\begin{array}{ccc} \lambda_{A} & = & \arcsin\frac{x}{N}, \end{array}$$

(11) $$\begin{array}{ccc} \phi_{A} & = & \arcsin\frac{y}{M}, \end{array}$$

where *x* and *y* are the horizontal and vertical integer numbers of pixels between projections of actually analysed point *A* denoted as $A_{x}$ and $A_{y}$ and the central point *C*.

Considering the above conditions, the distance function defined as:

(12) $$l_{A}=r\;\arccos\left(\cos\left(\arcsin\frac{y}{M}\right)\cos\left(\arcsin\frac{x}{N}\right)\right).$$

is proportional to the angles that define the longitude $\lambda_{A}$ and the latitude $\phi_{A}$ of the point *A* expressed by horizontal *x* and vertical *y* integer number of pixels calculated from the image center, respectively.

As previously, we assume the sphere to be a unit sphere, therefore r=1. Further, to obtain cos(•) function values, not the angles themselves, the function arccos(•) has also been left out.

Finally, the spatial version of the correction function is defined as:

(13) $$\gamma \left(x,y\right)=\cos\left(\arcsin\frac{y}{M}\right)\cos\left(\arcsin\frac{x}{N}\right).$$

The defined above function $\gamma \left(x,y\right)$ is then used to compute γ value for the gamma image correction procedure [25]. Usually, gamma correction is performed globally for the whole image with a fixed gamma value.

**Figure 3:**
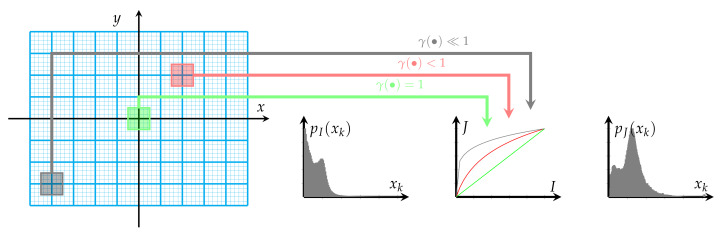
Overview of the proposed image enhancement depending on window spatial location.

Gamma correction is a nonlinear operation that allows the visibility of dark (γ<1) or bright (γ>1) image details to be enhanced, depending on the γ value. In the current study, the gamma correction procedure is performed not for the whole image but independently in small, square, non-overlapping windows of size w×w. The window size *w* is specified as the smallest possible square, yet larger than one pixel and, of size defined by the greatest common divisor (*gcd*) of the image size, i.e., w=gcd(2N,2M). The value of γ in each window is a mean value of γ(x,y) function in this window. This principle of the proposed brightness correction procedure is presented in Figure 3.

Centrally located window (green window in Figure 3 with x≈y≈0) corresponds to the gamma function value near 1, i.e., γ(x,y)≈1, thus in the resulting image these pixels’ intensities remain unchanged comparing to the original image. The farther away the window is from the image center, the smaller the value of the γ(•) function is (red and gray windows in Figure 3). It means that pixels in the output image are significantly brightened the farther the moving window from the image center is. To recapitulate, visibility, as well as contrast in originally dark peripheral areas of the analysed image, are improved.

As an initial image processing procedure, contrast and brightness enhancement are required for many operations during UWFA image processing and analysis. The presented approach is automatic, local, and parameterless. Therefore, it is beneficial over traditional, especially manual and global, image enhancement methods that require a proper choice of parameters.

### 2.3. Image Database

Medical examinations were carried out at the Department of Ophthalmology, Faculty of Medical Sciences in Zabrze, Medical University of Silesia, Katowice, Poland. The study was performed in adherence to the tenets of the Declaration of Helsinki and approved by the Ethics Committee of the Medical University of Silesia (decision KNW/0022/KB1/125/I/18/19). The written informed consent from the participants had been obtained. Among patient inclusion criteria were: age 18 or greater, diabetes mellitus type 1 or 2, nonproliferative diabetic retinopathy. Among the exclusion criteria were: retinal photocoagulation, history of pars plana vitrectomy or cataract surgery with posterior capsule rupture, media opacity disabling to assess the fundus of the eye, proliferative diabetic retinopathy, vitreoretinal traction in the macula, nonperfusion of the foveal area, any concurrent nondiabetic retinal disease. Patient preparation to image acquisition involved pupils dilation by administration of 1% tropicamide (Polpharma, Starogard Gdański, Poland) and phenylephrine 2.5% to the conjunctival sac followed by intravenous sodium fluorescein (250 mg; SERB, Paris, France) administration. A more detailed description of the patient cohort and the medical aspects are presented in [27].

Fluorescein angiography retinal images were taken using Optos California P200DTx (Optos, Dunfermline, UK) device during three phases [28]: early (E, up to 60 s), mid (M, from 1st up to the 5th min), and late (L, 5th min and above). The testing image database contains 256 UWFA images and is summarized in Table 1. The spatial resolution of each image is 3900 × 3072 pixel, 8-bit grayscale, and stored in .tif file format.

### 2.4. Quality Measures

In order to assess the method, we have used several image quality measures known from the literature. Among them are: Edge Based Contrast Measure (EBCM) [29,30], indices showing edge preservation properties—Noise Suppression (ρ) and Measure of the Edge Preservation (β) [31,32], measures based on the structural information of the image, i.e., measure of structural similarity (SSIM) [33], Quality Index *Q* [34], and the most straightforward parameters based on pixel-to-pixel error measurement, i.e., Mean Absolute Error (MAE), Mean Squared Error (MSE), Root Mean Square (RMS) [35]. Definitions of all the used measures are presented in Appendix A. Here only the expected values of the measures are summarized for clarity: (1) EBCM = 1—no change, EBCM > 1—contrast improvement, (2) ρ, β, SSIM, Q—the closer to 1 the better, the value of 1 achievable for identical images, (3) MAE, MSE, RMS—the closer to 0 the better, the value of 0 achievable for identical images.

## 3. Results

**Figure 4:**
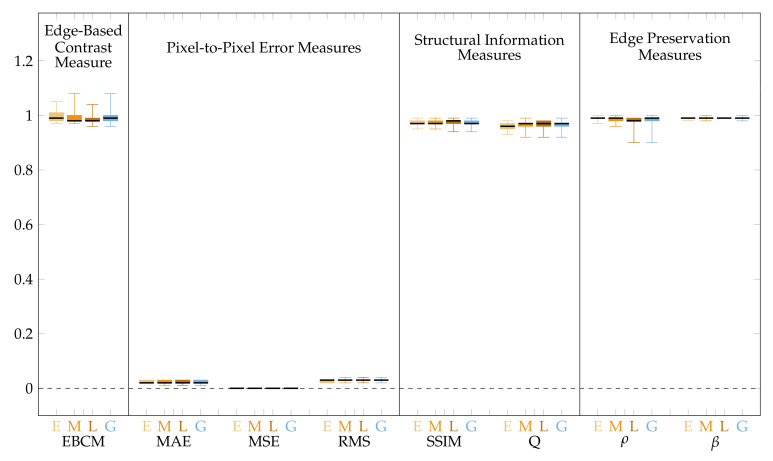
Quality measures for images processed by the proposed method. Measure values calculated from the central image regions only.

**Figure 5:**
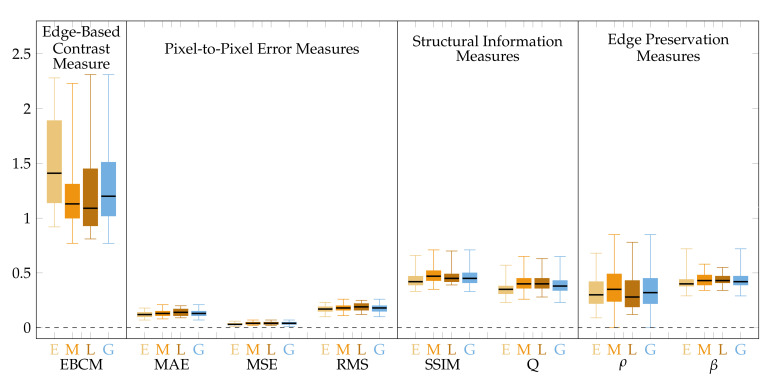
Quality measures for images processed by the proposed method. Measure values calculated for the peripheral image regions only.

Verification of the method was performed using the aforementioned image database (Section 2.3) considering the image acquisition phases (E, M, L) and globally (G) for all of the 256 available UWFA images. Images were divided between the acquisition phases since images with different timings may exhibit overall image brightness and contrast differences. The assessment was performed separately for the central and peripheral parts of the images. The image area is thus divided into 9 subregions with borders at 25% and 75% of width and height of the image. The measures are then calculated separately for the central (Figure 4) and combined peripheral (Figure 5) regions. The results are presented using boxplots grouped in the four categories of image quality measures. Boxplot colours correspond to the image acquisition phase categories from Table 1.

**Figure 6:**
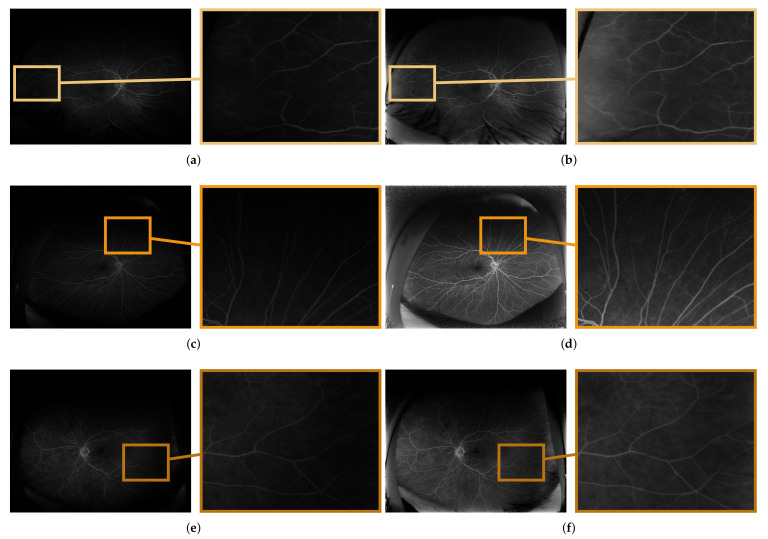
Comparison of exemplary UWFA images: before (**a**,**c**,**e**) and after (**b**,**d**,**f**) the correction procedure; images taken from the: (**a**,**b**) early, (**c**,**d**) mid and (**e**,**f**) late acquisition phases.

The qualitative performance of the proposed methodology is presented on exemplary images taken from the database. The images before and after the correction procedure are shown in Figure 6. Selected regions are enlarged for clarity to present the method’s performance. The quality measures for the selected regions from Figure 6 are given in Table 2 as well.

Because the CLAHE (Contrast Limited Adaptive Histogram Equalization [15,25]) is one of the most frequently used algorithms for image preprocessing in retinal angiography [16,17,18,19,20,21], it was also included for comparison. The assessment was performed using the same approach and quality measures as for the proposed method. For both methods, the same window size was used (the image was divided into the same number of rows and columns—’Number of tiles’—to get a window of 12 × 12 pixels). All remaining parameters of the CLAHE method were left with default values [15].

**Figure 7:**
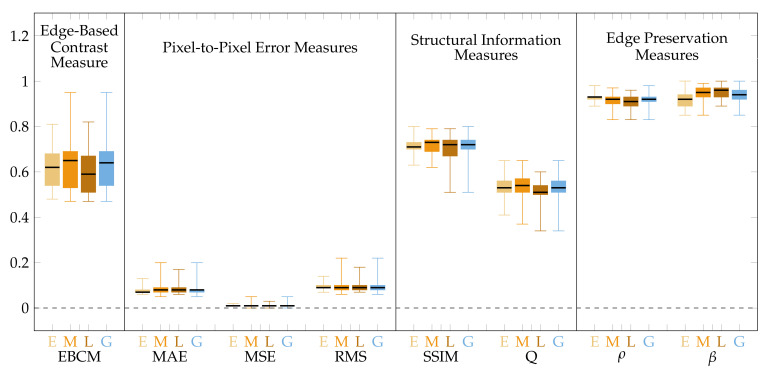
Quality measures for images processed by the CLAHE method.Measure values calculated for the central image regions only.

**Figure 8:**
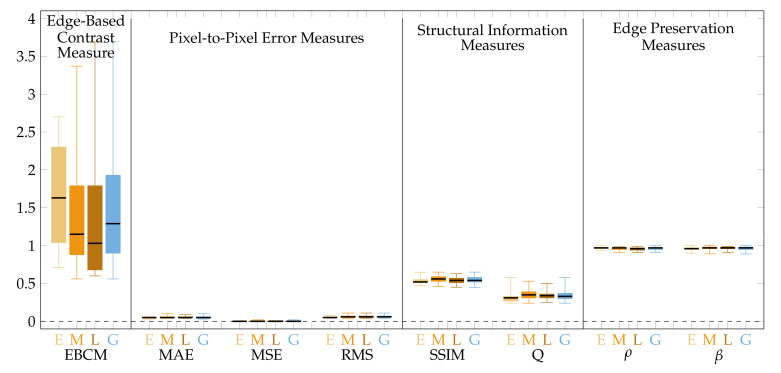
Quality measures for images processed by the CLAHE method. Measure values calculated for the peripheral image regions only.

The obtained for CLAHE quality measure values are presented graphically in Figure 7 for the image central regions and in Figure 8 for the peripheral regions.

Average values of the quality measures for the central and peripheral regions for both proposed and CLAHE methods are presented in Table 3.

**Figure 9:**
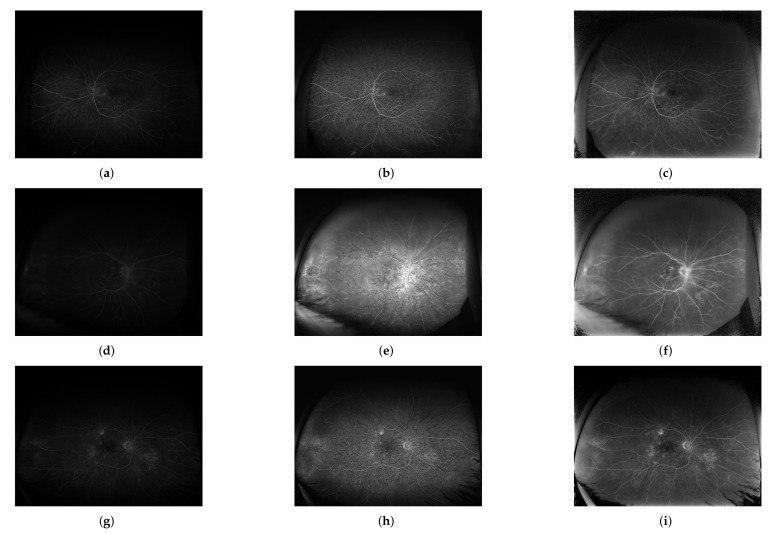
Visual comparison of the results obtained for several input images (**a**,**d**,**g**) by the CLAHE method (**b**,**e**,**h**) and the proposed method (**c**,**f**,**i**) for three phases: early (first row), mid (middle row) and late (last row).

Visual inspection and comparison of the resulting images obtained using both approaches are shown in Figure 9 on three exemplary images from the early, mid, and late acquisition phases.

## 4. Discussion and Conclusions

For the proposed method, as can be seen from the Figure 4 and Table 3, the quality measures obtained from the central image region are close to 1 (EBCM, SSIM, Q, ρ and β). This indicates that the proposed processing method does not affect the central image region much. The pixel-to-pixel measures are clustered around 0, pointing that the method does not introduce errors (noise) to the images in the central regions. The average values of all the measures are very similar within each of the four categories.

From Figure 5 and Table 3, it can be noticed that the quality measures for the peripheral region are much more dispersed. EBCM by average is >1, indicating that the resulting image can be considered better in contrast and edge visibility. SSIM and Q are about 0.47 and 0.38, respectively. Those measures, however, take into account the average change in luminance between the images [34], thus the lower value of those measures is expected since the average luminance in the peripheral regions is increased. It is worth noting that the median values of the structural information measures are very similar between all four image groups. Moreover, the distributions of EBCM and SSIM measures are not symmetrical, with more cases above the median what is preferable. The ρ coefficient appears to be the most dispersed. As a measure aimed at the assessment of noise suppression [32], it indicates that in the peripheral regions, noise may be introduced. The metric takes into consideration the average image intensity level, therefore its value is expected to be lower than for the central regions. The ρ and β measures for the central areas take values close to 1, indicating a negligible difference between the compared images. On the other hand, the values are lower for the peripheral areas, which shows the difference between the compared images. The mentioned differences indicate the edge enhancement in the resultant image.

**Figure 10:**
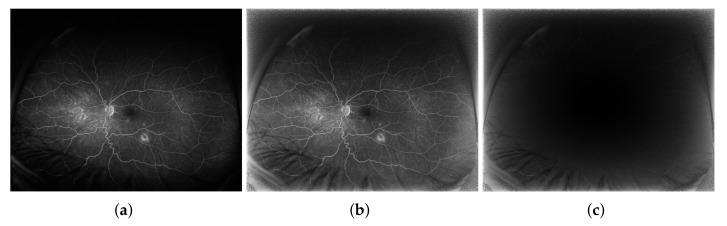
Error distribution for pixel-to-pixel measures: (**a**) exemplary original image, (**b**) contrast enhanced image, (**c**) AE values for each image pixel.

The pixel-to-pixel measures values are higher than the corresponding values in the central image regions yet are still close to 0, which shows that the method does not introduce significant errors (noise) to the images in the peripheral regions. The distribution of one of the pixel-to-pixel measures—Absolute Error (AE, that is MAE before averaging)—is presented in Figure 10, which shows a certain image before (Figure 10a) and after (Figure 10b) being subjected to the contrast enhanced procedure and the AE values for each image pixel (Figure 10c). The AE values are small for pixels located in the image center. The further away the pixel is from the image center, the larger the coefficient value is. The highest values are achieved in the corners, where no diagnostically important information is expected.

The CLAHE method does not consider spatial relations in the image, therefore the processing is performed in the same manner in all image regions, which contrasts with the presented approach. CLAHE influences central image regions more, which is indicated by worse values of all the quality measures compared to the proposed method (Figure 7, Table 3). Those regions exhibit high brightness variability (blind spot as a region of high intensity surrounded by a relatively dark background). In extreme cases, e.g., for underexposed images, there is brightness saturation after CLAHE application (Figure 9e). The proposed method does not influence the central image regions that much, which is shown by the value of EBCM measure of about unity and is visible in Figure 9f.

In the peripheral image regions characterized by much lower contrast and overall brightness, the influence of the CLAHE approach is lower than the proposed method. It is shown by the measures from the pixel-to-pixel group (MAE, MSE, RMS) that for the CLAHE are lower than for the presented approach (Figure 8, Table 3). The edge preservation measures (ρ and β) seem better for the CLAHE approach as the measures are close to unity. Those measures for the proposed algorithm are of lower value because the brightness is enhanced more than the contrast. It also can be seen in Figure 9b vs. Figure 9c and Figure 9e vs. Figure 9f. The EBCM values obtained for both methods also support the observation. Structural information indices (SSIM and Q) are comparable for both processing algorithms, with SSIM being slightly higher and Q being a bit lower for the CLAHE processed images compared to the proposed method.

For underexposed images (like Figure 9d) the CLAHE approach causes saturation of central image regions and border artifacts along the vasculature (Figure 9e). The presented method does not introduce such issues as can be seen in Figure 9f. This is advantageous in cases of not correctly acquired images because the screening does not have to be repeated for the patient. Moreover, in some cases (especially visible in Figure 9h) the CLAHE algorithm causes an over sharpening of the image exposing local disturbances and influencing the background perception. The image can be perceived as more granular than the original, therefore, the diagnosis about leakages and microaneurysms may be influenced. The proposed method does not sharpen the image, thus does not introduce high frequency noise. At the same time the details in the peripheral regions are comparable to those visible after application of the CLAHE algorithm.

Summarised interpretation of the obtained quality measures’ values is presented in Table 4. The expected behaviour in the central image regions is minimal image distortion (EBCM ≈ 1, MAE, MSE, RMS ≈ 0, SSIM, Q, ρ, β≈ 1 indicate minimal image modification). Correspondingly the expected behaviour in the peripheral regions is image enhancement (EBCM > 1, MAE, MSE, RMS > 0, SSIM, Q, ρ, β<1 indicate that the output image differs from the input image, the larger distance from 1 the more the image is affected).

To conclude, the advantage over the CLAHE approach of the proposed method is in brightness enhancement resulting in more even intensity distribution over the whole retina image without the extra sharpening. The technique might be beneficial for underexposed images. Last but not least, the proposed approach does not require the setting of any controlling parameters. Therefore it is easy to be applied, especially by inexperienced technicians. At the same time, it does not exclude the application of more advanced processing methods if required in clinical practice for specific cases.

## 5. Summary and Future Work

The paper presents a straightforward, geometry-based and parameterless method for UWFA image preprocessing. The method assumes that the eyeball is spherical, and the correction depends on the distance from the centre. The contrast enhancement is done locally in non-overlapping blocks with the gamma correction method, in which the gamma value is calculated automatically. The method was assessed and compared to the commonly used CLAHE algorithm using several image quality measures that show its performance and usefulness in UWFA image processing.

**Figure 11:**
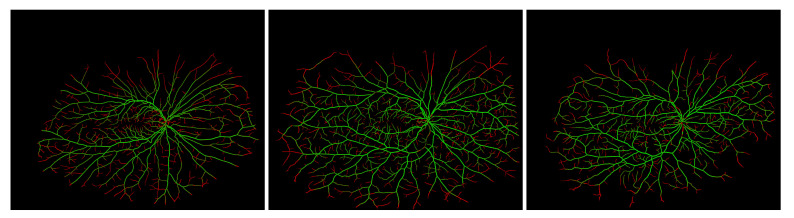
Exemplary vasculature segmentation results on images without the preprocessing by the proposed method (green) and after being processed by the proposed method (red).

The proposed UWFA image preprocessing was also applied during a pilot study dedicated to retinal blood vessels segmentation. The experiment consisted of vascular segmentation from original and brightness-enhanced images obtained by the presented approach. To detect the blood vessels, an algorithm optimized for UWFA images was adopted [36]. The database consisted of 12 UWFA images supplemented with ground truth vasculature segmentation. Exemplary segmentation outcomes for three medical cases are presented in Figure 11. The results obtained without the brightness compensation are shown in green and in red—when the presented enhancement method was applied. As can be seen, the employment of the presented preprocessing method enables the segmentation algorithm to detect more peripheral vasculature and small vessels in the whole image area. Those outcomes are promising for future works.

## Figures and Tables

**Table 1:** Summary of the image test database.

Subjects	Eye		Images		Phase		
	L	R	L	R	Early	Mid	Late
34	19	22	124	132	74	146	36
	41		256			256	

**Table 2:** Quality measure values for selected windows from Figure 6.

Phase	EBCM	MAE	MSE	RMS	SSIM	Q	ρ	β
Early	0.97	0.1	0.02	0.13	0.67	0.61	0.04	0.66
Mid	0.97	0.03	0.00	0.03	0.94	0.92	0.99	0.99
Late	0.99	0.05	0.00	0.05	0.84	0.80	0.83	0.95

**Table 3:** Average values of quality measures obtained from the central and peripheral image regions for processed by the proposed and CLAHE methods.

Measure	Method	Central Region				Peripheral Region			
		E	M	L	G	E	M	L	G
EBCM	Proposed	1.00	0.99	0.99	0.99	1.51	1.21	1.24	1.30
	CLAHE	0.62	0.62	0.59	0.62	1.88	1.46	1.99	1.57
MAE	Proposed	0.02	0.02	0.02	0.02	1.12	0.13	0.14	0.13
	CLAHE	0.08	0.08	0.08	0.08	0.05	0.06	0.06	0.05
MSE	Proposed	0.00	0.00	0.00	0.00	0.03	0.04	0.04	0.04
	CLAHE	0.01	0.01	0.01	0.01	0.00	0.00	0.00	0.00
RMS	Proposed	0.03	0.03	0.03	0.03	0.17	0.18	0.19	0.18
	CLAHE	0.09	0.10	0.09	0.09	0.06	0.06	0.06	0.06
SSIM	Proposed	0.97	0.97	0.97	0.97	0.44	0.49	0.48	0.47
	CLAHE	0.71	0.72	0.70	0.71	0.53	0.56	0.54	0.55
Q	Proposed	0.96	0.97	0.97	0.97	0.35	0.41	0.41	0.39
	CLAHE	0.53	0.54	0.51	0.53	0.31	0.36	0.34	0.34
ρ	Proposed	0.99	0.99	0.98	0.99	0.33	0.37	0.31	0.35
	CLAHE	0.93	0.92	0.91	0.92	0.97	0.96	0.96	0.96
β	Proposed	0.99	0.99	0.99	0.99	0.41	0.44	0.44	0.43
	CLAHE	0.92	0.95	0.95	0.94	0.96	0.96	0.97	0.96

**Table 4:** Interpretation of quality measures values for UWFA image brightness enhancement: “+” in favor of the presented method, “−” in favor of the reference method, “∘” if methods are comparable. Fields within parentheses are additionally discussed in the text.

Image Region	EBCM	MAE	MSE	RMS	SSIM	Q	ρ	β
Central	+	+	∘	+	+	+	+	+
Peripheral	(−)	+	+	+	(∘/+)	∘	(+)	(+)

## Data Availability

The image data could be provided on request after contact with B.S.-M.

## Appendix A. Quality Measures

The proposed methodology is primarily dedicated to improve peripheral retina areas in UWFA images. However, this improvement cannot worsen sharpness of the image, degrade its structural information, increase noise level, and last but not least, adversely affect the contrast and location of edges. Considering these conditions, the evaluation of the method is performed using metrics classified into several groups characterized below. Among them are measures assessing contrast enhancement [29,30] as well as indices satisfying edge preservation properties. Additionally, the most straightforward parameters based on pixel-to-pixel error measurement (i.e., MAE, MSE, NMSE, RMS) as well as measures based on the structural information of the image (i.e., SSIM, Quality index) are also evaluated. Definitions of all the used measures are presented in the following sections.

### Appendix A.1. Edge-Based Contrast Measure

The Edge-Based Contrast Measure (EBCM) stems from the observation that the human perception mechanisms are very sensitive to contours (or edges) [29]. The gray level corresponding to object frontiers is obtained by computing the average value of the pixel gray levels weighted by their edge values. Contrast $C(p_{i})$ for a pixel $p_{i}$ of image *I* is thus defined as:

(A1) $$C\left(p_{i}\right)=\frac{|I\left(p_{i}\right)-E\left(p_{i}\right)|}{|I\left(p_{i}\right)+E\left(p_{i}\right)|},$$

where E(•) is the mean edge gray level given by

(A2) $$E\left(p_{i}\right)=\frac{\sum_{p_{j}\in N\left(p_{i}\right)}g\left(p_{j}\right)\cdot I\left(p_{j}\right)}{\sum_{p_{j}\in N\left(p_{i}\right)}g\left(p_{j}\right)},$$

$N(p_{i})$ is the set of all neighboring pixels $p_{j}$ of pixel $p_{i}$, and $g(p_{j})$ is the edge value at pixel $p_{j}$. Without loss of generality, we employ 3 × 3 neighborhood, and $g(p_{j})$ is the magnitude of the image gradient estimated using the Sobel operators [25]. The EBCM for image *I* is thus computed as the average contrast value

(A3) $$EBCM\left(I\right)=\frac{\sum_{p_{i}\in D}C\left(p_{i}\right)}{\left|D\right|},$$

where *D* is the image domain (a set of all pixels $p_{i}$), |D| is the number of pixels in the image.

For an output image *J* obtained from an input image *I*, it is expected that the contrast is improved when the condition EBCM(J)≥EBCM(I) is satisfied [30]. In this work we used the relative value of this measure, defined as:

(A4) $$EBCM\left(I,J\right)=\frac{EBCM(J)}{EBCM(I)}$$

therefore values larger than 1 indicate image contrast improvement.

### Appendix A.2. Pixel-to-Pixel Error Measures

In order to evaluate the noise reduction level, conventional, simple, and widely used quality metrics are used.

The first metric is the Mean Absolute Error (MAE):

(A5) $$MAE\left(I,J\right)=\frac{1}{\left|D\right|}\sum_{p_{i}\in D}\left|I\left(p_{i}\right)-J\left(p_{i}\right)\right|,$$

which is a measure of differences (errors) between paired observations expressing the same phenomenon. In the current approach it is a difference between the reference I(•) and resulted J(•) images, where *D* is the image domain (a set of all pixels $p_{i}$) and |D| is the cardinality of the image domain (number of pixels in the image).

This category of measures also includes the Root Mean Square (RMS) defined as the standard deviation of the pixel intensities [35]:

(A6) $$RMS=\sqrt{\frac{1}{\left|D\right|}\sum_{p_{i}\in D}{\left(I\left(p_{i}\right)-J\left(p_{i}\right)\right)}^{2}}.$$

Similar in nature to MAE is the distortion index, called Mean Squared Error (MSE), which is expressed as:

(A7) $$MSE\left(I,J\right)=\frac{1}{\left|D\right|}\sum_{p_{i}\in D}{\left(I\left(p_{i}\right)-J\left(p_{i}\right)\right)}^{2}.$$

Small values of the measures mentioned above indicate small difference between the images being compared, therefore values close to zero are expected.

### Appendix A.3. Structural Information Measures

Due to strong spatial dependencies of image pixels, Wang et al. [33] introduced a measure of structural similarity (SSIM) as an alternative framework for quality assessment based on the degradation of structural information under the assumption that human visual perception is highly adapted for extracting structural information from a scene [37].

The SSIM is defined as:

(A8) $$SSIM\left(I,J\right)=\frac{\left(2\mu_{I}\mu_{J}+C_{1}\right)\left(2\sigma_{IJ}+C_{2}\right)}{\left(\mu_{I}^{2}+\mu_{J}^{2}+C_{1}\right)\left(\sigma_{I}^{2}+\sigma_{J}^{2}+C_{2}\right)},$$

where $C_{1}={\left(\left(L-1\right)\cdot k_{1}\right)}^{2}$, $C_{2}={\left(\left(L-1\right)\cdot k_{2}\right)}^{2}$, L=256, $k_{1},k_{2}\ll 1$,

(A9) $$\mu_{I}=\frac{1}{\left|D\right|}\sum_{p_{i}\in D}I\left(p_{i}\right),\;\mu_{J}=\frac{1}{\left|D\right|}\sum_{p_{i}\in D}J\left(p_{i}\right),$$

(A10) $$\begin{array}{ccc} \sigma_{I}^{2} & = & \frac{1}{|D|-1}\sum_{p_{i}\in D}{\left(I\left(p_{i}\right)-\mu_{I}\right)}^{2},\\ \sigma_{J}^{2} & = & \frac{1}{|D|-1}\sum_{p_{i}\in D}{\left(J\left(p_{i}\right)-\mu_{J}\right)}^{2}, \end{array}$$

and

(A11) $$\sigma_{IJ}=\frac{1}{|D|-1}\sum_{p_{i}\in D}\left(I\left(p_{i}\right)-\mu_{I}\right)\left(J\left(p_{i}\right)-\mu_{J}\right).$$

The resultant SSIM index is a value between −1 and 1, and value 1 is only reachable in the case of two identical sets of data (images).

Another structural information measure called the Quality Index *Q* was defined by Wang et al. [34] as:

(A12) $$Q=\frac{4\sigma_{IJ}\mu_{I}\mu_{J}}{\left(\sigma_{I}^{2}+\sigma_{J}^{2}\right)\left(\mu_{I}^{2}+\mu_{J}^{2}\right)}$$

The dynamic range of *Q* index is [−1;1], the closer the value is to 1 the better.

### Appendix A.4. Edge Preservation Measures

The aforementioned indexes do not reflect the edge preservation, so further measures are introduced [32]. The first factor is a measure of the Noise Suppression (ρ). It is based on correlation and is defined as:

(A13) $$\rho =\frac{\Gamma (I-\bar{I},J-\bar{J})}{\sqrt{\Gamma \left(I-\bar{I},I-\bar{I}\right)\cdot \Gamma \left(J-\bar{J},J-\bar{J}\right)}}$$

where $\bar{I}$ and $\bar{J}$ are mean values in *I* and *J*, respectively and

(A14) $$\Gamma \left(s_{1},s_{2}\right)=\sum_{p_{i}\in D}s_{1}\left(p_{i}\right)\cdot s_{2}\left(p_{i}\right).$$

The second is a Measure of the Edge Preservation (β), defined as:

(A15) $$\beta =\frac{\Gamma (\Delta I-\bar{\Delta I},\Delta J-\bar{\Delta J})}{\sqrt{\Gamma \left(\Delta I-\bar{\Delta I},\Delta I-\bar{\Delta I}\right)\Gamma \left(\Delta J-\bar{\Delta J},\Delta J-\bar{\Delta J}\right)}},$$

where ΔI and ΔJ are a highpass filtered versions of *I* and *J*, respectively, obtained with a 3×3 standard approximation of the Laplacian operator, while $\bar{\Delta I}$ and $\bar{\Delta J}$ are mean values of ΔI and ΔJ, respectively [31,32]. The correlation measures defined by Equations (A13) and (A15) should be high, close to 1, when the estimated image is similar to the reference image.
